# The role of DLL1 in long-term vascular normalization and cancer immunotherapy

**DOI:** 10.20892/j.issn.2095-3941.2021.0507

**Published:** 2021-10-29

**Authors:** Qiaozhen Wu, Yuhui Huang

**Affiliations:** 1 Suzhou Ninth Hospital Affiliated to Soochow University, Suzhou 215200, China; 2 Hematology Center, Cyrus Tang Medical Institute, The Collaborative Innovation Center of Hematology, State Key Laboratory of Radiation Medicine and Protection, Soochow University, Suzhou 215123, China

Cancer immunotherapy, especially immune checkpoint blockade therapy (ICB), has revolutionized cancer treatment, providing a potential curable modality for advanced cancer patients. In immunogenic cancer, such as melanoma and lung cancers with a history of smoking, approximately 30% patients with anti-programmed death 1 (PD1) monotherapy survive more than 5 years. However, despite being a major breakthrough in the treatment of cancer, the majority of cancer patients have not yet benefited from ICB monotherapy^1^. Thus, innovative strategies to improve ICB efficacy are still needed.

**Figure 1:**
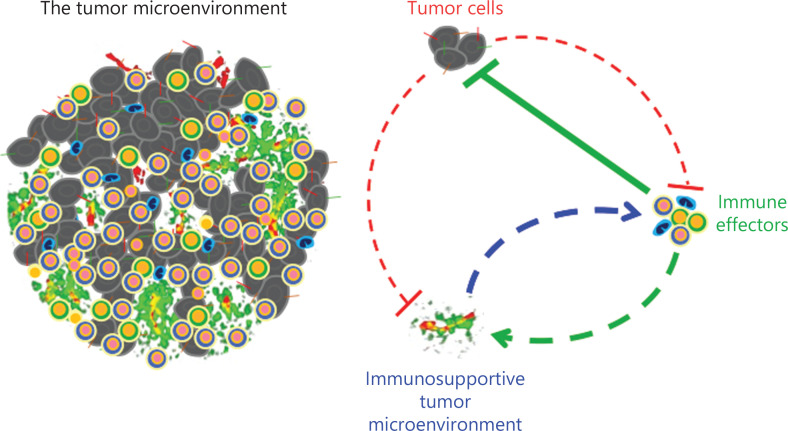
Three prerequisites for successful cancer immunotherapy. Tumor cells secrete various factors that often suppress immune effector functions and promote the formation of an aberrant tumor microenvironment (TME). Tumor antigens are targets for immune effectors to distinguish malignant cells from normal cells. Immune effectors can recognize tumor antigens and destroy malignant cells. The TME provides a location for immune effectors to perform their functions. In addition, activated immune effectors can recondition the TME to become more favorable to antitumor immune responses. Therefore, sufficient tumor antigens, functional immune effectors, and an immune-supportive TME are the prerequisites for a successful cancer immunotherapy. The feedback interactions between immune effector activation and TME normalization are crucial in eliciting potent and durable antitumor immunities to eradicate malignant cells.

Conventional cancer treatments, such as chemotherapy and targeted therapy, control tumor progression by directly killing malignant cells. In contrast, cancer immunotherapy eliminates malignant cells by stimulation of tumor-antigen specific immune responses. Thus, a successful cancer immunotherapeutic approach needs 3 prerequisites (**Figure 1**): i) a tumor antigen is required for immune effectors to distinguish malignant cells from normal cells. Thus, immunogenic cancers usually have a higher abundance of tumor antigens and respond better to immunotherapy, when compared to low or nonimmunogenic cancers; ii) the presence of functional immune effectors, which recognize and destroy malignant cells. “Hot tumors” often exhibit more favorable prognoses than “cold tumors”, which lack immune effectors; iii) the presence of an immuno-supportive tumor microenvironment (TME), which is necessary for immune effectors to perform their antitumor functions. The TME is usually hypoxic and immunosuppressive, which hinders activation of immune effectors and antitumor immune responses. Multiple studies have reported that remodeling TME to create an immuno-stimulatory environment potentiated cancer immunotherapy^2^. In addition, activated immune effectors can normalize tumor blood vessels to alleviate immunosuppression in the TME, which further facilitates stimulations of immune effectors^3^. The interactions between tumor immunogenicity, immune effectors, and the TME therefore form a complex biological system, which regulates antitumor immunity, and may form the basis for the development of innovative therapeutic strategies to improve response rates and prolong patient survival.

Extensive efforts have been devoted to the study of tumor antigens and immune effectors, but the critical role of the TME in cancer immunotherapy remained unrecognized until the 2000’s. The TME contains many low oxygenation regions and is often enriched with inhibitory immune cells, which are a major obstacle for effective cancer immunotherapy. The abnormal profile of the TME is mainly due to the presence of aberrant tumor blood vessels^2,4^. These vessels are tortuous, dilated, and leaky with low pericyte coverage, resulting in tumor tissue hypoxia. Hypoxia promotes the accumulation of suppressive immune cells, such as regulatory T cells (Tregs) and myeloid-derived suppressor cells (MDSCs), and also polarizes immune cells from a pro-inflammatory to an anti-inflammatory phenotype, such as M1-*vs.* M2-like tumor-associated macrophages. The alleviation of tumor tissue hypoxia is therefore a promising way to overcome the immunosuppressive TME and to enhance cancer immunotherapy^4,5^. Targeting of proangiogenic factors, such as vascular endothelial growth factor (VEGF) signaling, can normalize tumor blood vessels, reduce hypoxia, and increase tumor-infiltrating CD8^+^ T cells while reducing Tregs and MDSCs, resulting in better therapeutic efficacies^4,6^. However, the vascular normalization window with current available anti-angiogenic therapies is transient, which limits its benefit for cancer immunotherapy. Simultaneously targeting multiple proangiogenic factors or calibrating dosage could improve tumor vascular normalization, although the effects are still temporal. Therefore, novel strategies are needed to generate long-term vascular normalization of tumors.

Notch signaling is evolutionarily conserved and plays crucial roles in both embryogenesis and tissue differentiation, including cell fate decisions, immunity, and angiogenesis. Notch signaling consists of 4 receptors, Notch 1−4, and 5 ligands involving Delta Like Canonical Notch Ligand 1 (DLL1), DLL3, DLL4, Jagged 1, and Jagged 2^7^. Among them, DLL1, DLL4, and Jagged 1 have been implicated in blood vessel formation. Blockade of DLL4 inhibits tumor growth, even in tumors with intrinsic resistance to VEGF inhibitors, indicating the important roles of Notch signaling in tumor angiogenesis^8^. Notably, anti-DLL4 treatments induce non-functional tumor blood vessel growth, with its chronic intervention leading to hemangioma^9^. In contrast, elevated DLL1 levels in the TME decrease tumor vessel density, increase vessel perfusion, and reduce tumor tissue hypoxia, when compared to control tumors, suggesting that DLL1 induces tumor vascular normalization^10^. Moreover, functional blood vessels are evenly distributed across the entire tumor parenchyma in tumors with elevated expressions of DLL1. These effects are also significant in large tumors (8−9 mm), corresponding to 3 weeks of post-tumor inoculations. Together, these results show that elevated DLL1 levels in the TME induce long-term tumor vascular normalization^10^. As we mentioned above, a successful cancer immunotherapeutic regimen requires tumor antigen, activated immune effectors, and an immuno-supportive TME. Elevated DLL1 levels in the TME normalize tumor blood vessels, and also promote the infiltration and activation of CD8^+^ T cells, thus in combination with anti-CTLA4 antibody treatments, they reverse tumor therapy resistance and significantly prolong survival, when compared to monotherapy intervention^10^. Conversely, DLL1 knockout in dendritic cells impedes antigen-specific CD8^+^ T cell activation, reduces interferon γ (IFNγ) production, and facilitates the growth of lung and pancreatic tumors^11^. DLL1/Notch signaling is therefore a potential novel target to induce tumor vascular normalization and to improve cancer immunotherapy^7,10^.

Why can selective activation of DLL1/Notch signaling induce long-term tumor vascular normalization? Normal blood vessel formation is regulated by the homeostasis of pro- and anti-angiogenic factors^12^. The relentless production of pro-angiogenic factors due to uncontrolled tumor cell proliferation breaks this balance and prompts tumor angiogenesis. Accordingly, inhibitors of pro-angiogenic factors can reverse this imbalance and induce tumor vascular normalization. Unfortunately, the duration of vascular normalization is approximately 2−8 days in preclinical tumor models, and the clinical benefit of antiangiogenic treatments involving chemotherapy is usually in the order of weeks^12^. Overall, the initial blockade of pro-angiogenic factors reduces vessel density and retards tumor growth, but only to relatively lower levels. Under these circumstances, continuous treatment often increases tumor tissue hypoxia, inducing the production of different pro-angiogenic factors. Consequently, drug resistance rapidly develops, and the vessel normalization effects disappear. Targeting pro-angiogenic factors is therefore unlikely to permanently restore homeostasis of pro- and anti-angiogenic factors, because it can be easily disrupted by persistent secretion of pro-angiogenic factors in the tumor parenchyma. From this perspective, we propose an innovative strategy for long-term suppression of tumor angiogenesis and induction of tumor vascular normalization by modulation of angiostatic factors. Angiostatic factors are a group of physiologically occurring anti-angiogenic factors, but tumors generally do not increase the production of these factors. Thus, intervention to elevate angiostatic factors antagonizes the effects of pro-angiogenic factors, which may not induce drug resistance, and vessel normalizing effects may last longer. Moreover, durable vascular normalization reduces tumor tissue hypoxia, leading to the reduction of pro-angiogenic factor production, which further facilitates vascular normalization. This positive feedback loop improves and prolongs tumor vascular normalization. During increases of DLL1 levels, long-term tumor vascular normalization is induced by CD8^+^ T cells and IFNγ. IFNγ is an angiostactic factor, which successfully overcomes the effects of proangiogenic factors. Thus, IFNγ-mediated tumor vascular normalization could be durable when the levels of IFNγ are not too high.

Consistent with the vascular normalizing effects of angiostatic factors, our previous studies showed that immune checkpoint therapy activated CD8^+^ T cells, elevated IFNγ production, and induced tumor vascular normalization, which only occurred in tumors sensitive to treatments. Moreover, increased vessel perfusion upon immune checkpoint therapy, the major characteristics of tumor vascular normalization, is positively correlated with the efficacies of treatments^3^. Based on these results, we proposed a new mechanism for immune checkpoint therapy: immune checkpoint inhibitors activate T cells, which normalizes tumor blood vessels, converting the immuno-suppressive TME to an immuno-permissive TME. This, in turn, facilitates the expansion and improves the functions of intratumoral effector T cells. Such a positive feedback loop involving immune-vascular crosstalk reinforces antitumor immunity and vascular normalization, resulting in long-term tumor control^4^. Thus, knowledge of immune-vascular crosstalk may represent a novel way to gain additional insight into the mechanisms of tumor immune evasion, tumor angiogenesis, and the aberrant TME, which could provide the basis for the development of more effective and efficient strategies to treat cancer patients. For example, because improved tumor vascular function is positively correlated with the efficacy of immune checkpoint therapy, investigators may study whether the responsiveness of a tumor to immune checkpoint inhibitors can be predicted by monitoring tumor vessel functions. Because vascular functions can be monitored in real time by noninvasive radiological methods, tumor vascular function can possibly be used as a novel noninvasive biomarker to design personalized cancer immunotherapies^3,4^.
